# Factors Associated with Non-Participation and Non-Adherence in Directly Observed Mass Drug Administration for Malaria in The Gambia

**DOI:** 10.1371/journal.pone.0148627

**Published:** 2016-02-11

**Authors:** Susan Dierickx, Charlotte Gryseels, Julia Mwesigwa, Sarah O’Neill, Melanie Bannister-Tyrell, Maya Ronse, Fatou Jaiteh, René Gerrets, Umberto D’Alessandro, Koen Peeters Grietens

**Affiliations:** 1 Department of Public Health, Institute of Tropical Medicine, Antwerp, Belgium; 2 Amsterdam Institute of Social Science Research, Amsterdam, The Netherlands; 3 Rhea, Centre of Expertise Gender, Diversity and Intersectionality, Vrije Universiteit Brussel, Brussels, Belgium; 4 Medical Research Council Unit, Fajara, The Gambia; 5 London School of Tropical Medicine and Hygiene, London, Unoted Kingdom; 6 Partners for Applied Social Sciences (PASS) International, Tessenderlo, Belgium; 7 School of Tropical Medicine and Global Health, Nagasaki University, Nagasaki, Japan; Johns Hopkins Bloomberg School of Public Health, UNITED STATES

## Abstract

**Introduction:**

The potential benefits of Mass Drug Administration (MDA) for malaria elimination are being considered in several malaria endemic countries where a decline in malaria transmission has been reported. For this strategy to work, it is important that a large proportion of the target population participates, requiring an in-depth understanding of factors that may affect participation and adherence to MDA programs.

**Methodology:**

This social science study was ancillary to a one-round directly observed MDA campaign with dihydroartemisinin-piperaquine, carried out in 12 villages in rural Gambia between June and August 2014. The social science study employed a mixed-methods approach combining qualitative methods (participant observation and in-depth interviewing) and quantitative methods (structured follow-up interviews among non-participating and non-adhering community members).

**Results:**

Of 3942 people registered in the study villages, 67.9% adhered to the three consecutive daily doses. For the remaining villagers, 12.6% did not attend the screening, 3.5% was not eligible and 16% did not adhere to the treatment schedule. The main barriers for non-participation and adherence were long and short-term mobility of individuals and specific subgroups, perceived adverse drug reactions and rumors, inconveniences related to the logistics of MDA (e.g. waiting times) and the perceived lack of information about MDA.

**Conclusion:**

While, there was no fundamental resistance from the target communities, adherence was 67.9%. This shows the necessity of understanding local perceptions and barriers to increase its effectiveness. Moreover, certain of the constraining factors were socio-spatially clustered which might prove problematic since focal areas of residual malaria transmission may remain allowing malaria to spread to adjacent areas where transmission had been temporarily interrupted.

## Introduction

Approximately one-third of malaria endemic countries are pursuing strategies to achieve malaria elimination [1–3]. However, attaining such an ambitious goal may not be feasible with standard control strategies, e.g. Long-Lasting Insecticidal Nets (LLIN), Indoor Residual Spraying (IRS) or larval source management [2,4]. Moreover, in low endemic countries and seasonal malaria settings, a large proportion of *Plasmodium falciparum* infections are carried asymptomatically and in low densities. Asymptomatic infected individuals, mostly undetectable by microscopy or rapid diagnostic tests (RDT), can maintain transmission, particularly where malaria transmission is low [1,5]. Mass drug administration (MDA), treating all individuals regardless of their infection status, addresses this problem [1,5,6]. In addition, where malaria transmission is seasonal, it is started every year by asymptomatically infected individuals who in turn infect the vectors. By clearing such infections, MDA has the potential of preventing the yearly malaria peak [1,2,6,7], which could have a greater impact than just treating malaria patients attending health facilities [1,6,8]. Furthermore, as malaria risk is heterogeneous, resulting in pockets of transmission [9], MDA could be used to target these “hotspots” [10]. MDA is currently the control or elimination strategy for other neglected tropical diseases (e.g. lymphatic filariasis, schistosomiasis, and onchocerciasis), despite controversy surrounding the effectiveness and acceptability of such a strategy [1,2,7,11–16].

The effectiveness of MDA for malaria control or elimination, may be compromised by the difficulty of achieving sufficient coverage either due to refusals or by poor treatment adherence [1,5,6,8]. There are few reports and studies on factors influencing the participation in, and adherence to, MDA campaigns targeting malaria [17]. The aim of this study was to explore the motivations and circumstances for non-uptake and non-adherence of MDA with dihydroartemisinin-piperaquine (DHAPQ) (Eurartesim^®^) in rural Gambia.

## Methods

### Study site and population

The anthropological study was ancillary to a study looking at the dynamics of malaria transmission and at factors determining its heterogeneity (referred to hereafter as the ‘Malaria Transmission Dynamics study’). Twelve rural villages located in five regions of The Gambia, namely West Coast River, North Bank, Lower River, Central River and Upper River Regions (North Bank and South Bank), were included in the MDA campaign and provided the study setting for the anthropological study. The selection of the study villages for the MDA campaign is described in Mwesigwa et al. [18]. It can be summarized as follows: it is based on a nationwide school survey and a follow-up malariametric survey in six villages surrounding the school with highest seroprevalence of anti-malarial antibodies in each region. In 2013, the village with the lowest and highest surrounding prevalence for each region were selected to be part of the Malaria Transmission Dynamics study.

As part of this study inhabitants were actively followed up by monthly bleedings during the rainy season carried out by fieldworkers and passive case detection at local health facilities. The MRCG fieldworkers collected a blood sample for the detection of malaria infections by PCR, and in case of symptomatic individuals a rapid diagnostic test (RDT) for the immediate detection of a malaria infection was performed.

Malaria transmission in The Gambia is highly seasonal (August to December) [9,19]. The total population of the study villages was about 4,500 people, mainly *Mandinka*, *Serahule*, *Wolof* and *Fula*, with the large majority being Muslim and practicing subsistence farming. Remittances from family members living in Europe and America contributed to the livelihoods of some families. Villages comprised compounds containing several households related by kinship.

### The implementation of MDA in the study setting

One round of directly observed MDA with DHAPQ (Eurartesim^®^)—a drugs with a prophylactic functioning—was carried out from June to August 2014, before the beginning of the malaria transmission season. It was preceded by sensitization meetings to inform the communities of the MDA intervention and personal written consent. All DHAPQ doses were directly observed for the three-day treatment by a team of nurses and fieldworkers from the Medical Research Council Unit, The Gambia (MRCG), assisted by local village health workers (VHW) and community volunteers. Individuals with confirmed positive RDT, documented cardiac or renal pathological conditions, pregnant women (all women ≥15 years old were systematically screened with a urine pregnancy test), infants < 5 kg, and adults > 75 years old were excluded from the MDA. Participants with uncomplicated malaria and pregnant women were referred to the government health facilities for the appropriate treatment; underweight children and people > 75 years did not receive an alternative drug. This was the first MDA carried out in these study villages.

### Study design

A mixed-methods design was used for the social science study.

During the first phase, qualitative data were collected for independent analysis and to prepare the quantitative strand. During the first qualitative strand, ethnographic research was conducted in all study villages, with focused fieldwork during the MDA in two pairs of adjacent villages (Njayel and Madina Samako in the Upper River Region south bank; Sare Wuro and Gunjur Koto in the Upper River Region north bank). These two pairs of villages were theoretically selected to be observed during MDA, based on expected (i) lower participation/adherence to the treatment and (ii) higher human mobility. Literature indicated the importance of these variables for the success of MDA, and previous qualitative research by the researchers had shown the presence of these factors in the four selected villages.

During the second strand of research, quantitative data were collected to confirm and quantify results from the qualitative component. The quantitative phase was initiated when the list documenting MDA uptake became available. Quantitative data were systematically collected by administering a questionnaire among non-participating (did not take any medication) or non-adhering (did not take all 3 doses) individuals in all twelve villages.

### Qualitative data

#### Data collection

Participant observation consisted of participating in everyday activities in the communities and observing the sensitization and drug distribution during MDA. Reiterated informal conversations with MRCG fieldworkers and community members, regardless of their decision to participate, were carried out, leading to an in-depth understanding of more sensitive topics such as social relationships and trust in MDA and the MRCG.

Interviews with community members were recorded and fully transcribed. These interviews were carried out before, during and directly after MDA. When not possible and/or inappropriate given the sensitive nature of the topic discussed, the conversation was not recorded immediately but its content was written down immediately after the interview. Interviews were mostly carried out in the local languages, namely *Madinka*, *Fula* and *Serahule*. The question guide focused on factors influencing MDA such as the informed consent procedures, mobility, adverse drug reactions and perceptions of the trial.

#### Sampling

Following the principle of gradual selection, informants were theoretically selected (in accordance with emerging results/theory) and categorized in relation to relevant criteria (e.g. gender, age, socio-economic status, decision to participate and adhere). In addition, “snowball” sampling techniques—sampling using participants to identify additional respondents—were used in order to increase confidentiality with respondents and consequent reliability of the data [20].

#### Data analysis

In accordance with the research strategy, data analysis was a flexible and iterative process. Preliminary data from the observations, informal conversations and interviews were collected and analyzed to inform the interview guide; in-depth interviews were then conducted to confirm or refute temporary results until saturation was reached. Interviews were systemized and analyzed with NVivo 10 Qualitative Analysis Software (QSR International Pty Ltd. Cardigan UK).

### Quantitative data

#### Data collection

Following the MDA, a questionnaire was administered to adults who did not participate (did not go for screening) or did not complete the treatment (intake of 1 or 2 doses), focusing on potential barriers for participation and adherence. The questionnaire consisted of a combination of open- and closed-ended questions.

#### Sampling

The sampling frame consisted of adult community members who did not participate or adhere to the MDA as recorded by in the MDA trial registration list. People who were not allowed to participate due to the MDA exclusion criteria were not included. Preliminary data of the MDA trial registration list showed that 63.7% of the people who did not take the medication or did not adhere were minors, and 36.3% were adults. Every 4^th^ adult person on the list was selected, and respondents were re-visited 3 times before exclusion from the quantitative study. Seventy-four adults from all villages were systematically selected from the trial registration list that documented non-participation/non-adherence.

#### Data analysis

Quantitative data were double entered in Microsoft Excel and cleaned and analyzed in SPSS (IBM SPSS Statistics 22). Frequency tables and cross-tables for the main outcome variables were produced.

### Ethical clearance

The Malaria Transmission Dynamics study, including MDA, received ethical approval by the MRC Scientific Coordinating Committee and The Gambia Government/MRC joint Ethics Committee (SCC number 1318). The social science study was approved by the MRC Scientific Coordinating Committee and The Gambia Government/MRC joint Ethics Committee (SCC number 1319) and by the Institutional Review Board of the Institute of Tropical Medicine, Antwerp, Belgium. The interviewers followed the Code of Ethics of the American Anthropological Association (AAA). People were informed about the social science research in community sensitization meetings preparing the overall Malaria Transmission Dynamics study. Before participating in the social science research, respondents were informed about project goals, the topic and type of questions as well as their right to decline participation or to interrupt the conversation at any time. Anonymity was guaranteed and confidentiality of interviewees assured by assigning a unique code number to each informant. Verbal instead of written consent was preferred as requesting the subject’s signature could have been a potential reason for mistrust.

## Results

### Participation and adherence to MDA

Among the 3942 people (including children, adults and elders) registered in the study villages, 87.4% (3445/3942) participated (agreed to be screened) and 67.9% (2675/3942) adhered to the three consecutive doses (Table 1).

**Table 1:** Participation and adherence to MDA among study population (N = 3942).

	n	%
Did not want to participate (not screened for MDA)	497	12.6
Did want to participate (screened for MDA)	3445	87.4
*- Intake of 1 dose*	189	4.8
*- Intake of 2 doses*	442	11.2
*- Intake of 3 doses*	2675	67.9
*- Not eligible*	139	3.5

Among the 3445 people screened for the MDA, 96% (3306/3445) was eligible to participate [4% was not eligible (139/3445)]. 77.7% (2675/3445) adhered to the three consecutive DHPAQ doses while [18.3% (631/3445) did not (intake of 1 or 2 doses)] (Table 2).

**Table 2:** Adherence to MDA among people screened (N = 3445).

	n	%
- Intake of 1 dose	189	5.5
- Intake of 2 doses	442	12.8
- Intake of 3 doses	2675	77.7
- Not eligible	139	4.0

A total of 74 adult individuals were included in the quantitative study, 49 of them had refused the MDA and 25 had not completed the 3-day treatment (Table 3). The large majority of them were of *Fula* ethnicity (60.6%) and women (56.8%) were slightly more represented (Table 4).

**Table 3:** Participation and adherence to MDA among study participants quantitative strand (N = 74).

	n	%
Intake of 0 doses	49	66.2
Intake of 1 dose	12	16.2
Intake of 2 doses	13	17.6

**Table 4:** Socio-demographic characteristics of study participants quantitative strand (N = 74).

	n	%
**Gender**		
Male	32	45.9
Female	42	56.8
**Ethnicity**		
Fula	45	60.6
Madinka	8	10.8
Jola	5	6.8
Tilibonka	4	5.4
Serahule	3	4.1
Other ethnic groups	5	8.2
Missing information	3	4.1

### Reasons for non-participation or adherence

The main barriers for non-participation and non-adherence were long and short-term mobility of individuals and specific subgroups, perceived adverse drug reactions and related rumors, inconveniences related to the logistics of MDA (e.g. waiting times) and the perceived lack of information about MDA (Tables 5 and 6).

**Table 5:** Reasons for non-participation in MDA (multiple answers possible) (N = 49).

	n	%
Mobility	23	32.9
Perceived side effects	8	11.4
Lack of trust	13	18.6
Waiting time	13	18.6
Drug characteristics	2	2.9
Public pregnancy test	2	2.9
Misunderstanding MDA	5	7.1
Compound/Household head did not allow	3	4.2
Missing information	1	1.4
Total answers	70	100

**Table 6:** Reasons non-adherence to MDA (multiple answers possible) (N = 25).

	n	%
Mobility	7	20
Perceived side effects	15	42.8
Waiting time	4	11.4
Drug characteristics	1	2.9
Public pregnancy test	2	5.7
Misunderstanding MDA	4	11.4
Compound/Household head did not allow	1	2.9
Missing information	1	2.9
Total answers	35	100

### Mobility

The most common reason for non-participation was human mobility (32.9%) (Table 5). Among non-adherent respondents this was the second major barrier for full participation (20%) (Table 6). Mobility was understood as long term, seasonal or routine human population movements over considerable distance between and within countries, hence it does not include people working on the farms or staying at schools who could have reached the MDA distribution point. The qualitative study indicated that different mobility patterns influenced MDA in distinct ways. Potential participants were (i) absent during the MDA; (ii) not documented in the baseline census and consequently not actively identified during the MDA; (iii) absent during community sensitization meetings; and, (iv) temporarily not residing in the villages. Specific mobility patterns were: (i) cross-border movements between The Gambia and Senegal, such as people working or studying in Senegal, but frequently returning to their home village in The Gambia, or Senegalese citizens migrating temporarily to work on farms in The Gambia during the rainy season; (ii) rural to urban movements for temporary and permanent work and health care, particularly along the Gambian coast; (iii) rural to rural movements, such as men looking for job opportunities in the agricultural sector just before and during the rainy season; and, (iv) cattle herding, especially among male *Fula* herders in search of grazing land for their cattle.

### Perceived side effects

While fear of perceived side effects was a factor influencing initial participation (11.4%) (Table 5), it was the most important barrier for non-adherence (42.8%) (Table 6). The most commonly reported perceived side effects were fever, stomach pain, skin rashes, diarrhea, dizziness, fatigue (or general malaise) and vomiting. Some people interpreted these as normal effects of antimalarials, demonstrating DHAPQ ‘worked’. Coping mechanisms for perceived minor side effects included taking the medicine during the evening so symptoms would appear during sleep or simply stopping the treatment.

*‘When I took the drugs it affects me a lot because when I am in bed it seems like the bed is moving*. *It caused certain things that also my mother had never experienced before*. *And I had headache*, *vomiting and high temperature*. *It took me two days to recover*, *I did not go out so due to that*, *I took the drug once*.*’*

(Housewife, Njayel)

As the MDA was conducted at the beginning of the rainy season during intense agricultural activity, the perceived side effects would also lead to non-adherence as people feared it prevented them from working in the fields.

Respondents rarely mentioned serious side effects, e.g. extreme tiredness, loss of consciousness and ‘talking nonsense’ (hallucinations), as reaction to MDA. When people did mention serious side effects, qualitative data showed that participants and their family members stopped taking the required doses. People experiencing side effects did not always contact the MRCG nurses for medical care.

### Trust

For 18.6% of non-participating respondents, the lack of trust in the MDA campaign was a barrier for participation (Table 5). Interestingly, it does not show in the quantitative data about the non-adherent populations. Nevertheless, qualitative data indicates that upcoming rumors regarding the side effects had an effect on people’s adherence. Qualitative data showed that fears about the side effects turned into rumors regarding the safety of the drug affecting the uptake of MDA in some compounds in certain villages. Examples of elements that fueled fears were rumors that the drug was still in trial; the MDA team did not take the drugs themselves, and the requirement to sign or thumb print the informed consent sheet which was perceived not to safeguard people’s interests but to assure that the MRCG could not be held responsible in case of serious adverse events.

*‘The only skepticism that I have is that you want to give me medication and you want me to sign*. *In case I do sign for it and a very bad adverse reaction happens to me*, *how can I manage that because I have already accepted taking the pills*?*’*

(Male farmer, Bessi)

Some people were distrustful about the intentions of the MRCG because they feared the recurrent blood sampling required for the main Malaria Transmission Dynamics study (once per month during the previous 6 months of the rainy season). Previous frictions and political disputes among different compounds within the village would exacerbate discussions around the safety of the drug. Few female respondents reported they were not able to participate (4.2%) (Table 5) or adhere (5.7%) (Table 6) because their compound head or husband did not allow them. Qualitative research showed that the disapproval of the compound head or husband was also a constraining factor for children and adolescents. This disapproval was usually due to pre-existing disagreements with the MDA team or a general distrust towards to the MRCG. Nevertheless, most community members trusted the MRCG MDA fieldworkers as they had resided and provided free health care in the study villages during the previous malaria season.

### Inconveniences related to MDA procedures

For 18.6% of respondents, waiting time was a barrier for participation (Table 5); and for 11.4% of the respondents it influenced their decision to not complete the medication (Table 6). Observations revealed that the procedure of consenting, having their body weight and temperature measured, and, when relevant, a pregnancy test and a RDT performed before receiving the first drug dose, resulted in long waiting times, and in some villages to discussions about who should receive the drugs first: whether it should be children who had to go to school or men herding their cattle or farming the land. In addition, because treatment had to be taken on an empty stomach, some individuals were asked to return home and come back later, regardless of the time they had been waiting. MDA participants, especially children, complained about the size and the bitter taste of DHAPQ tablets which made it difficult to swallow, and consequently failed to participate or adhere (Tables 5 and 6).

Finally, qualitative data showed that most pregnant women did not come for screening. In The Gambia, women intentionally conceal their pregnancies in order to avoid gossip and evil spirits. In particular, pregnant adolescents, unmarried women and older women, who feel shameful of their pregnancies due to their social position, would prefer not to participate. Despite the assurance that pregnancy testing was carried out privately, some women preferred not to go or to disrupt their screening (Table 5).

### Misunderstanding MDA

The large majority of non-participating (36.7%) (Table 7) and non-adhering individuals (52%) (Table 8) thought the MDA aimed at preventing malaria (Tables 5 and 6). Certain respondents mentioned during the interview that MDA aimed at curing or preventing diseases such as tuberculosis, asthma, heart disease, hypertension and general health problems. Other people stated they did not know they were eligible to participate.

**Table 7:** Knowledge about objective MDA of non-participants MDA (N = 49).

	n	%
Objective MDA		
Improve health	11	22.5
Treat sickness	1	2
Prevention of sickness	8	16.3
Prevention of malaria	18	36.7
Treat malaria	4	8.2
I don't know	7	14.3

**Table 8:** Knowledge about objective MDA of non-adherents to MDA (N = 25).

	n	%
Objective MDA		
Improve health	2	8
Prevention of sickness	8	32
Prevention of malaria	13	52
Treat malaria	1	4
I don't know	1	4

## Discussion

While several studies have looked into community factors relating to MDA for neglected tropical diseases [12,14,15], less is known about the potential barriers at community level for MDA with the objective of interrupting malaria transmission [7]. With a growing interest in MDA for malaria in elimination settings, there is the need to understand factors that affect the different implementation stages: screening, participation and adherence. Within the Malaria Transmission Dynamics study, MDA reached 67.9% of the study population after accounting for people who did not go for screening, were not eligible and those that did not adhere (Table 1).

This research shows that a single way of implementing MDA has variable results in different communities. As Krentel & Augner [14] discussed previously on *lymphatic filariasis* elimination, willingness to participate and to adhere to different drug doses is a highly situational decision. It is influenced by the interaction of demographic features, local health perceptions, the organization of MDA and other contextual factors. While some of these elements are health related (local health perceptions, fears surrounding pregnancy and drug characteristics), other factors are not. Non-health related factors seemed to be more prevalent and important for decision-making in the Gambian context and included mobility, trust and rumors, trial logistical factors, misunderstandings about MDA and the influence of the compound head/husband on the decision-making process.

The implicit assumption in many interventions is that rural villages are static entities, and as such mobile people and groups are often excluded from health interventions, explicitly because they do not conform to inclusion criteria (living permanently in a village) or implicitly because they are not present during the intervention. The exclusion of these mobile people and groups in MDA is problematic since they constitute a potential source of infection [21,22].

As reported in other contexts, the process of informed consent does not always achieve participants’ understanding of the trial in order to make a free and informed decision [23]. Although MRCG staff organized community meetings, and individual informed consent was obtained from study participants, people who did not participate or did not adhere, did not always understand the MDA’s objectives and procedures.

The origin and circulation of rumors, and how to target them, requires contextualized knowledge, especially for intervention targeting asymptomatic carriers. Taking medication when you are not sick requires trust in the institutions implementing the public health campaign [24–26]. The MRCG has been present in The Gambia for almost 70 years, and many people benefited of it. Trust in the MDA strategy was therefore based on previous positive interactions with the institution (MRCG) and its staff that resided in the study villages. Fieldworkers interact on a daily basis with researchers and community members [27] and their behavior is important for the success of the MDA. Their actions can limit MDA uptake, but they also can assist in enabling MDA by reassuring people and providing information in case of side effects. While the overall public trust in the MRCG is strong in the communities, a few people are skeptical about the MRCG apparently mostly due to fears surrounding recurrent blood sampling although detailed research on the generation of rumors and reasons behind them were outside the scope of this study. While, trust does not seem to directly influence people’s adherence based on the quantitative data, the small group of people who are skeptical about the MRCG, can have an influence on spread of rumors when side effects appear. Although the qualitative data demonstrated that side effects did lead to fear and rumors about the medication among the people who did not adhere, quantitative methods did not pick up on this sensitive topic as can be expected due to the formality of the data collection.

## Conclusion

The decision-making process to participate in MDA is influenced by a combination of health and especially non-health related factors that are highly heterogeneous and could consequently result in not covering a large social or spatial sub-group of the population. Even with a coverage of >80%, the spatial and social clustering of these sub-groups might allow focal areas of residual transmission to remain. Formative [28] social science research before, during and after MDA creates a better understanding of structural local constraints and concerns and can eventually provide the opportunity to contextualize the intervention and maximize potential participants’ free and informed decision to participation and adherence.
